# Linking Forest Ecosystem Services to the SDGs: Semi-quantitative Mapping of Perceptions towards Integrated Decision-making

**DOI:** 10.1007/s00267-023-01915-9

**Published:** 2023-12-04

**Authors:** Myriam Pham-Truffert, Jean-Laurent Pfund

**Affiliations:** 1 Earth System Science (ESS), Remote Sensing Laboratories, Department of Geography, University of Zurich, Zurich, Switzerland; 2 Centre for Development and Environment (CDE), University of Bern, Bern, Switzerland; 3 Digital Society Initiative (DSI), University of Zurich, Zurich, Switzerland; 4 Forest Division, Federal Office for the Environment (FOEN), Bern, Switzerland

**Keywords:** Forest ecosystem services, Sustainable development goals, Integrated landscape approaches, Policy integration

## Abstract

With this study, we test and present the results of a reproducible semi-quantitative methodological approach, which enables us to map perceptions of complex systems, linking the forest ecosystem services (FES) of a given spatial level to the wider policy domains represented by the 2030 Agenda and its Sustainable Development Goals (SDGs). Through a participative process, we used integrated forest management and FES as entry point concepts to support and inform dialog towards a normative desired future as framed by the SDGs, taking into account interdependencies across sectors and policy domains. The scales used in the test were national (Switzerland) and international but it is possible to use the approach at any level of integration, especially the landscape one in the case of forest or other ecosystem issues to be transdisciplinary solved. We stress that the semi-quantitative aspects of the approach – be it the ranking of the importance of FES across the different SDGs, or the positive or negative weighting of interactions among these FES in cross-impact matrices – enable the perceptions held by actors to be more explicit and significant for governance or goal prioritization. The results illustrate the perceptions of selected actors on the effects of integrated forest management and provide a basis for multi-actor deliberation on emerging potential synergies or conflicts, thereby genuinely supporting science-policy-practice dialog, which is crucial to foster integrated decision-making.

## Introduction

Since the Millennium Ecosystem Assessment, the concept of ecosystem services has demonstrated the complexity of ecosystems and the multiplicity of their (provisioning, regulating, supporting, and cultural) services (MA 2005). In the Swiss forest realm, the forestry framework in place has recognized for long forest functions, and therefore the multifunctionality of forest ecosystem services (FES) to society. FES is increasingly reflected in modern ways to consider the traditional forest management in Switzerland (Bernasconi et al. 2014), where regional participatory processes help evaluate the importance of forest functions in terms of sectoral planning. The concept of FES is also applied to forests globally, in particular with market-oriented conservation approaches involving payments for multifunctional FES (Landell-Mills et al. 2002; Wunder 2005).

While forests contribute notoriously to societal needs in terms of CO_2_ sequestration, biodiversity conservation, and livelihoods, the multifunctionality and the central role of forests for sustainable development *as a whole* often fails to reach beyond the community of forest experts and practitioners. Representatives of the forest realm often claim that forest goods and services are not yet sufficiently recognized and integrated into other sectoral policies and more generally in national or global political debates.

The 2030 Agenda for Sustainable Development which was adopted by all UN member states in September 2015 (UN 2015) provides a framework that can help embrace more complexity and communicate about multi-faceted benefits provided to society. Sustainable wellbeing (Costanza et al. 2016a; 2016b; Kubiszewski et al. 2021) is the overarching objective, as the agenda implies to reconcile human social development thresholds and the integrity of the biophysical life-support system (O’Neill et al. 2018).

With its 17 Sustainable Development Goals (SDGs) and 169 associated targets covering many social, economic and environmental dimensions of development, the 2030 Agenda is the current global framework for policy-making on sustainability issues. Since the outset of designing the SDGs, it was stressed that they needed to account for the systemic dynamics between potentially conflicting areas of development (Griggs et al. 2014; Le Blanc 2015; Renaud et al. 2022). For instance, ensuring energy supply (SDG 7) can clash with climate action (SDG 13) if the energy source generates emissions of greenhouse gases.

Through explicit considerations of the cross-sectoral and cross-scale nature of the dynamics underlying sustainable development, the 2030 Agenda has since then inspired efforts to mainstream a narrative of system thinking accounting for potential co-benefits to leverage and trade-offs to minimize (Kroll et al. 2019; Pham‐Truffert et al., 2020; Pradhan 2019; Anderson et al. 2021). Yet, there is a gap between the theory and the practice: While many SDG interaction studies and research have emerged in the scientific literature (see Bennich et al. 2020 and Horvath et al. 2022 for reviews of SDG interaction studies and Renaud et al. (2022) for an overview), cross-sectoral collaborations are far from generalized and the SDG domains are in practice still rarely addressed across different sectors. In that respect, Biermann et al. (2022) mostly observed discursive changes so far and stressed the limited profound normative and institutional changes since the adoption of the SDGs.

Thus, the call to better collaborate across sectors to use systemic synergies is still as important as ever both with regard to the SDGs, and also in light of the recognition of FES multifunctionality.

Integrated landscape approaches (ILAs) aim at reconciling biological conservation and the various land-uses implied by development (Reed et al. 2021), and suggest good practices to jointly address different social, economic and environmental objectives in complex adaptive systems. Among their ten principles for better anticipation and negotiation of future changes, Sayer et al. (2013) recommend in particular involving multiple actors fairly, addressing multiple scale issues, and reconciling multiple functions. Aligned with this proposition, our participative approach consists of semi-quantitative assessments – ranking of FES’ importance to the SDGs, and weighting of their interactions – enabling us to output explicit perceptions from the actors involved, i.e., their representation of how the system works. Such participatory mapping tools show great potential to facilitate integrated and inclusive landscape governance (Ros-Tonen et al. 2021), and are increasingly needed to produce relevant system knowledge (Magliocca et al. 2018) – which is especially crucial in landscapes with socially contested land claims (Meyfroidt et al. 2022).

This article contributes in concrete ways to better aligning with these principles by means of semi-quantitative data gathering. The objectives of this paper are: (1) to investigate the potential of linking the FES to the SDGs (2) to unravel complex dynamics underlying FES and the SDG domains with experts, and (3) to contribute more specifically to multi-actor dialog facilitation by testing a reproducible process to foster involvement into the decision-making process.

On the first point, research so far has mainly looked at the SDG contribution to forests rather than the other way round. For instance, a comprehensive scientific assessment of the potential effects of SDG implementation on forests has already been carried out by a large panel of international experts and scientists (Katila et al. 2019). More recently, a systematic literature review identified 63 SDG targets as having beneficial, damaging, or mixed impacts on forests (Carr et al. 2021). A few other studies already suggest linking the contribution of ecosystem services (Yang et al. 2020; Yin et al. 2021) or nature’s contributions to people (Anderson et al. 2019; Obrecht et al. 2021; Adhikari et al. 2022) to the SDGs. Further good examples of studies linking ecosystem services to the SDGs emerged from integrated approach in the fishery sector (Lynch et al. 2020) or in mountain forests (Gratzer and Keeton 2017). These services or contributions from nature are context-dependent and can be perceived as benefits or detriments to people (Díaz et al. 2018).

This leads to the second point, where the need to better understand co-benefits and trade-offs is stressed. In particular, we believe that considering the systemic influence of FES with one another is a way to ensure that the ecological factors of the system are not overlooked. Indeed, Reed et al. (2021) have shown concerns over a “rhetoric shifting” within the ILAs which overstate the synergetic relationships and tend to disregard the unavoidable trade-offs involved when considering different land uses. Regarding the relationships among these factors itself, most assessments of ecosystem service trade-offs and synergies fall short in considering the mechanisms or drivers (e.g., policy intervention or context) underpinning the relationships (Dade et al. 2019).

Finally, multi-actor dialog is key to understanding these relationships. With this study, we prompted forest-related experts to work with a cross-sectoral and integrated approach, embedding forestry priorities into the broader sustainable development agenda.

The remainder of this article is structured as follows. The methodology section first contextualize the forest management at scrutiny, leading in particular to a big picture of the history of the Swiss forestry, then elaborate on the data gathering and subsequent analysis. Next, the result section presents the FES’ contributions to the SDGs as well as the actors’ analytical understanding of the co-benefits and trade-offs arising amongst FES. Finally, the discussion part reflects on the applied method and on the contribution of our approach, and we conclude the article by stressing the main take-home messages.

## Methodology

### Contextualization

**Fig. 1:**
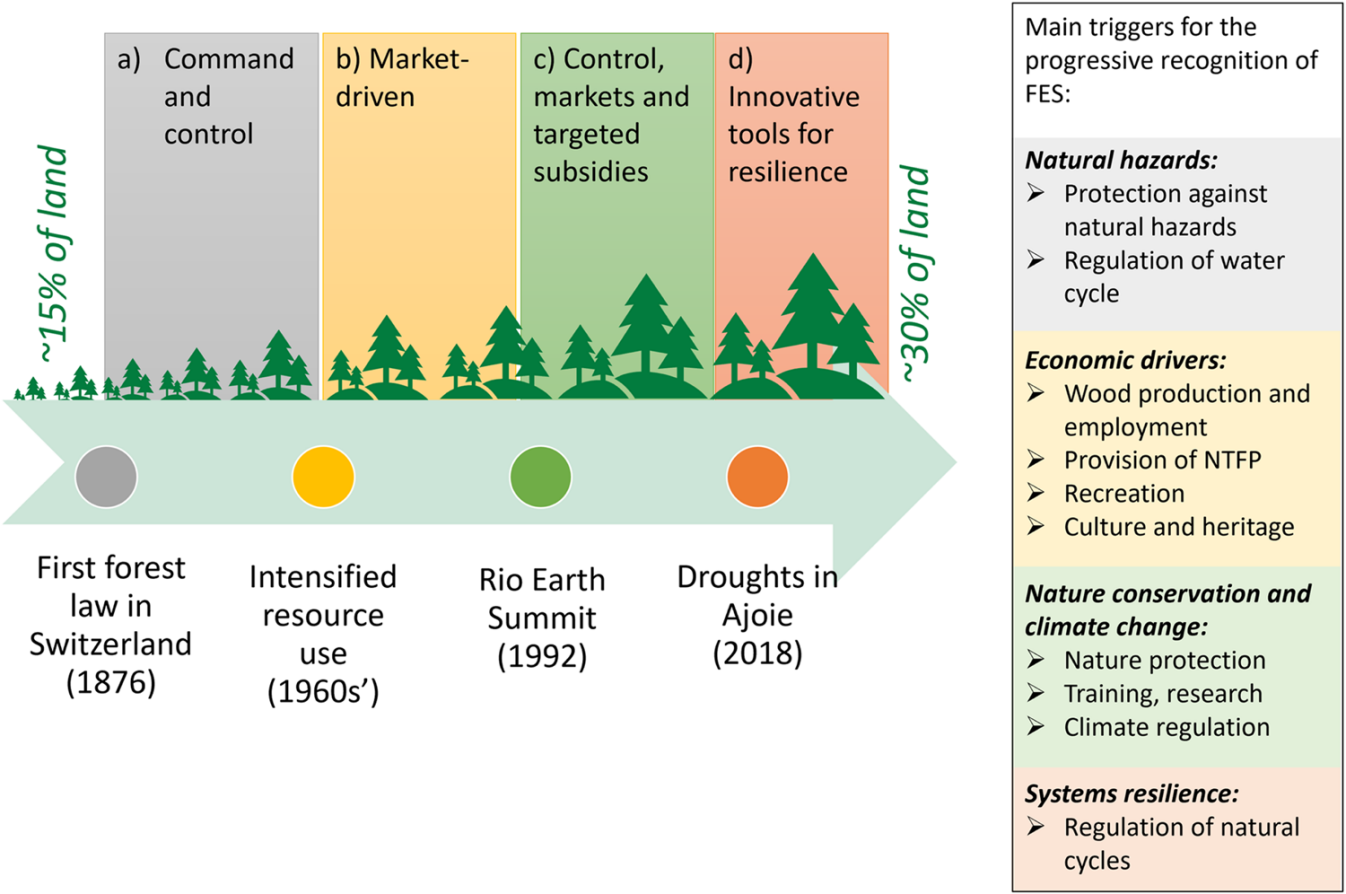
Main phases (**a**–**d**) of the Swiss forestry history

Context understanding is crucial and we provide in the following an account of the context that was also presented to the actors during the process. Based on a selection of pertinent papers and statements, we present a descriptive history of Swiss forestry and explain how and why Switzerland can be described as a country traditionally planning forest management in a rather integrated way (i.e., in a way that consider multiple FES) (Küchli 2013). This descriptive part is not an exhaustive literature review but relates to key documents providing the perspective of the Swiss forestry, further justifying the selection of the 10 FES by the historical development of forestry (see Fig. 1).

After several episodes of natural hazards linked to extensive clear-cutting of the Swiss Alpine forests (Capistrano et al. 2005), FES were acknowledged already back in the 19th century in Switzerland. The first Swiss forestry law, dating from 1876, explicitly states forests’ role to “serve as protection against climatic influences, wind damage, avalanches, falling rocks or ice, weakening of the ground, scouring, gullies and flooding” (Confédération Suisse (1876), authors’ translation). Forests’ ‘functions’ were defined, including for social welfare, and anchored in the Swiss Constitution. Most of them are now spatially defined for the whole country. In the 20th century, with the emergence of fossil fuels and electricity, wood consumption for energy purposes fell sharply and helped decrease the pressure on forests. Wood remained a valuable commercial good that long guided forestry along quantitative production principles. Coniferous species (i.e., spruce and fir) corresponded to market and transformation requirements and were either planted or promoted (Brändli 1998). The wood sector flourished and allowed the improvement of forest-related training and research as well as the consideration of all forest functions, including social ones, possible to include in the production-oriented system. However, notably in areas of low elevation, it was noticed that when the establishment of coniferous trees did not correspond to the ecological site conditions, issues of competition and diseases hampered foresters’ production objectives. Management strategies were considering species adaptation to local conditions and diversity as having an economic rationale. In parallel, starting in the ’70s-’80 s, political movements in favor of nature conservation were advocating for a consideration of its intrinsic values. The sustainable development concept coined in the Brundtland report (WCED 1987) helped to mainstream the idea that the environment and human development are inseparable and to conceptualize the balance to find between ecology, economy, and social needs.

Starting during the era of technical assistance in developing countries, a Swiss tradition of support for social forestry and sustainable management has taken place since the 1960s, ranging from field activities to international policy dialog. Swiss international aid now focuses, amongst others, on forest governance (Capistrano et al. 2005), as well as integrated forest management approaches (Aggestam et al. 2020). Integration amongst services and scales form crucial interest and competency areas for Switzerland to share internationally.

Nevertheless, the concept of integrated forest planning is not only challenged by a growing diversity of required FES but also by a rapidly changing environment. Swiss forests adapt to climate change to varying degrees, principally due to different management histories. Yet, practitioners and scientists observe on the ground previously unknown situations, such as rapid, drought-induced decrease of tree vitality in beech stands (Rohner et al. 2021). While diversified stands adapt to the new conditions, some remaining lowland monocultures, initially dedicated to wood production, suffer from pests and climatic effects. The severe impacts of the 2018–2019 droughts have been another important wake-up call for the Swiss forestry, leading to decentralized political interventions to adapt forests to climate change conditions. To this end, scientists suggest increasing the resistance, resilience and adaptability of forests – implying more tree species, structural and genetic diversity, and shorter turnover – with concrete adaptation measures such as planting, rejuvenating strokes, young forest maintenance, thinning or early use (Pluess et al. 2016).

### Data Collection

#### Selection of actors

Between September 2021 and October 2022, we tested our semi-quantitative approach by involving experts (n = 10) with relevant expertise and background to generalize how they perceived that FES contribute to the SDGs, and potentially foster or hinder each other at the relatively abstract Swiss and international levels.

To get a varied range of opinions and backgrounds, the experts were selected according to the diversity of their professional backgrounds as well as their expertise in integrated approaches: a national-level environmental NGO biologist (1), national and international experts from the federal administration (1 forester and 2 biologists) and from the cantonal level (1 forester), a national freelance forest engineer (1), a forest planning specialist (1), and two international forest scientists (2): one focusing on primary and secondary forests and the other on urban forest.

In addition to the knowledge of the Swiss forestry, the international experiences of the experts included fieldwork and yearslong projects conducted in forests in West Africa, Madagascar, South Africa, in South-East Asia, and in Eastern Europe among others.

#### Multiple FES

We built on the official text of the 2030 Agenda on Sustainable Development (UN 2015) and on the common international classification of ecosystem goods and services (CICES) (Haines-Young and Potschin 2010) and the system of environmental economic accounting (SEEA) ecosystem accounting (UN 2021) to establish two lists serving as a basis for the upcoming work. The first one was a full list of the 169 SDG targets with short and simplified descriptions (e.g., “renewable energy” for target 7.2); the second list consisted initially of 18 provisioning (5), regulating (8), and cultural (5) services through which forests could potentially contribute to the SDGs. These lists constituted a basis to instigate the forthcoming consultation of the afore-mentioned forest-related experts. Initially, the following FES were also listed: Access to land (reserve), provision of genetic resources, regulation of pests, soil regulation, spirituality and art, and spirituality, inspiration. However, these FES have been subsequently sub-categorized to classify under the 10 key FES listed below that were the closest to their meaning:

- Provision:
  - Wood production and employment
  - Provision of non-timber forest product (NTFP)
- Regulation:
  - Protection against natural hazards
  - Climate regulation
  - Regulation of water cycles
  - Regulation of natural cycles
- Cultural services:
  - Recreation
  - Training, research
  - Culture and heritage
  - Nature protection

Wood production and employment also include ”access to land (reserve)”, regulation of natural cycles include ”regulation of pest”, ”soil regulation” and ”provision of genetic resources”, culture and heritage include ”spirituality and art”, and recreation include ”spirituality, inspiration”.

#### Ranking of FES contributions to the SDGs

A first survey was sent in September 2021 to evaluate the level of importance of FES to each SDG at Swiss and international levels, with respectively five forest experts for each of the perspectives. Experts assessed the overall importance of FES contribution to each of the 17 SDGs, as well as the importance of specific services (i.e., aforementioned FES) to them, using a scale ranging from 0 (“No special relevance”) to +1 (important), to +2 (very important), and to +3 (crucial); For instance, the experts gave a score for the overall importance of forests’ contribution to SDG 5 on gender, then a score for each of the – in this case – three specific FES that the authors hypothesized as potentially relevant.

#### Identification of co-benefits and trade-offs

In the second stage, a second survey was sent to the same group of experts, who filled a cross-impact matrix on how the ten key FES impact each other positively, negatively, or both depending on the context (see de Jong et al. 2019). Practically, they assessed each of the 90 possible causal interactions as either positive (+1), negative (-1), context-dependent (+1 or -1), or not relevant (0).

### Data Analysis

To support actors’ dialog on forests’ contribution to the SDGs and integrated assessment of FES, we map the results of the surveys as directed, weighted links, in Sankey diagrams and network graphs respectively, which we discuss further in the results section.

#### Comparability

The survey results enable to see and compare two differentiated perspectives from the Swiss and international contexts. At these two levels, the assessment revealed the overall importance of forests’ contribution to each SDG and the contribution of specific FES to the given SDGs. The latter enabled us to go beyond the overall importance of forests’ contributions by SDGs and to foster discussions among the group of experts on tangible forests’ services and related activities.

#### Semi-quantitative aspect

At each level, we aggregated the survey results as mean scores of importance (ranging from 0 to 3) for the ranking of FES contibutions (first survey), and as sum of consensual assessment of a given interaction to be a trade-of or a synergy (second survey). For example, the interaction from nature protection to regulation of water cycles has been assessed to be a co-benefit by all five experts in charge of the international perspective, so the weight of this positive interaction is 5.

#### Workshops and dissemination

In the last stage, we organized a workshop to discuss and consolidate the results of the surveys. This step represented the negotiation phase and allowed people to openly discuss the results without coming back to their personal votes. This step allowed us to also identify the potential loose ends of our foreseen methodology and approach according to the full group of experts. A final step consisted in presenting the results and the overall process of our approach as an input at the World Forestry Congress (Pfund and Pham-Truffert 2022) and to the Interdepartmental Sustainable Development Committee Forest Subgroup (IDANE Wald + ) (Zabel et al. 2022) to showcase that it can support science-policy dialogs and integrated perspectives to policy and decision-making.

## Results

### Which Bundle of FES for which SDGs

**Fig. 2:**
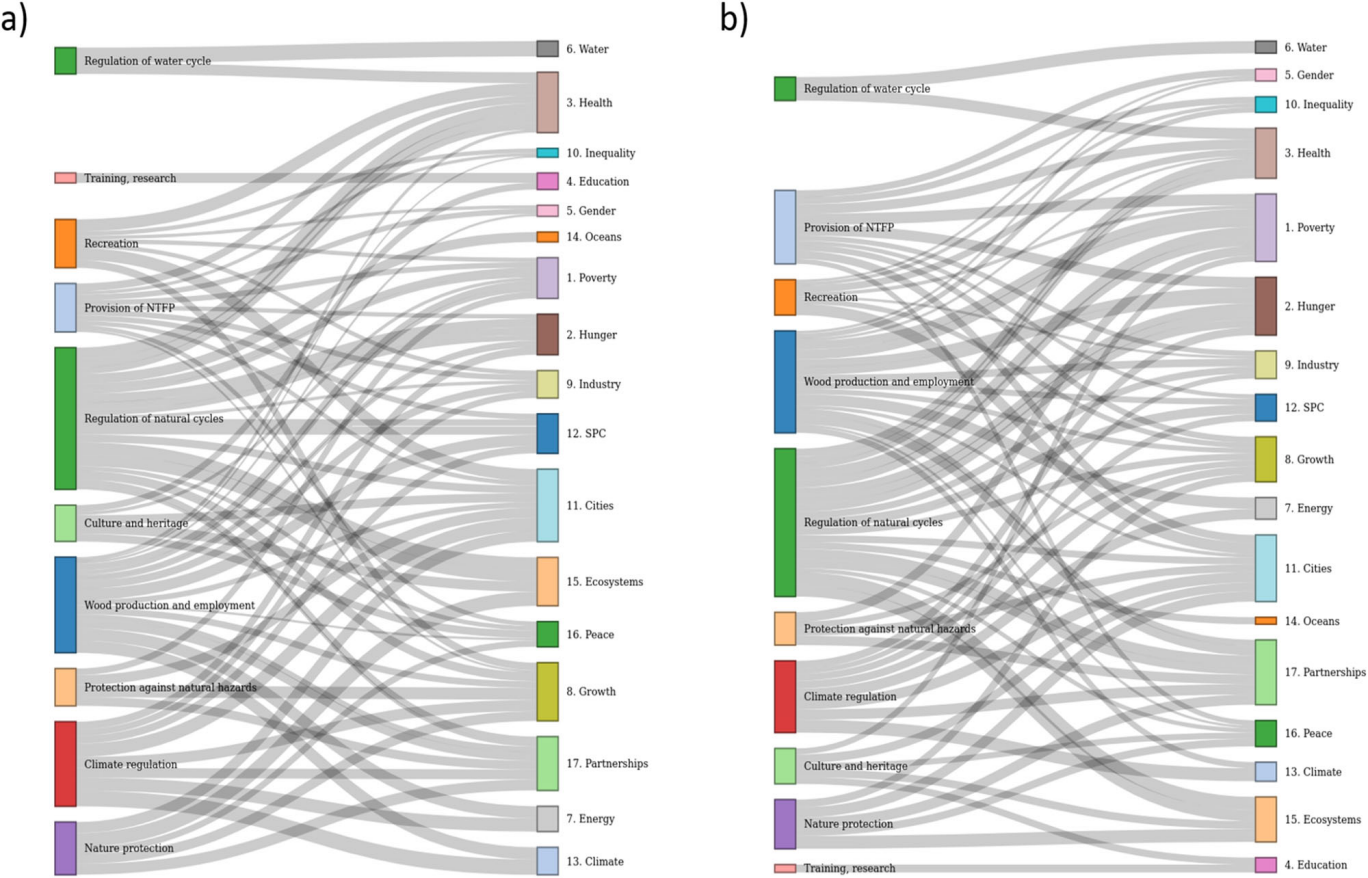
Importance of FES’ contribution to the SDGs in Swiss (**a**) and international (**b**) contexts. Links are the same but weights differ

One can first of all note that in Switzerland as well as globally, a majority of SDGs are covered by at least 5 different FES. Figure 2 displays in more detail the weighted importance of the ten specific FES’ contribution to the SDGs at Swiss and international levels as assessed by the group of experts.

The major difference between the assessed importance of FES’ contributions lies in the type of services being most valued depending on the context: Cultural services offered by forests (especially looking at recreation, as well as training and research) are generally assessed as more important in the Swiss than in the international context. Conversely, provision services, especially the provision of NTFP, are assessed as more important in the international context, notably contributing more to poverty and hunger alleviation (SDGs 1 and 2).

The first survey also enabled to assess the overall level of importance of FES’ contribution to each SDG according to the experts (see Table 1). As intuitively expected, the level of importance assessed for the goals related to water (SDG 6), climate (SDG 13) and terrestrial ecosystems (SDG 15) were the highest, both at Swiss (2.75/3) and international (2.67/3 for SDG 6 and 3/3 for SDGs 13 and 15) levels. A notable difference in the assessment at Swiss and international levels is observed for the good governance and partnerships’ goal (SDG 17) (1.25/3 in Switzerland versus 2.33 in the international context).

**Table 1:** Mean score for the overall importance to each SDG domain

SDG	Switzerland	International
1 (Poverty)	1.25	2.33
2 (Hunger)	1.25	1.67
3 (Health)	1.75	1.67
4 (Education)	1	1
5 (Gender)	0.5	0.33
6 (Water)	2.75	2.67
7 (Energy)	2	2.33
8 (Growth)	1	1.33
9 (Industry)	1	1
10 (Equality)	0.5	1
11 (Cities)	1.25	2
12 (SPC)	1.25	1.33
13 (Climate)	2.75	3
14 (Oceans)	1.5	1
15 (Ecosystems)	2.75	3
16 (Peace)	0.75	1.33
17 (Partnership)	1.25	2.33

In Switzerland, two types of forest contributions to SDG 7, related to energy, are then mentioned as “very important” (2/3): both wood production and climate regulation effects. Ranked close after those contributions, Swiss experts highlighted the link between forests and health through recreational use (1.75/3). The group of experts gave lesser importance (1.25/3) to forests’ contributions to the other goals, such as poverty, hunger, cities, sustainable production and consumption (SPC) and partnerships (1.25/3, for SDGs 1, 2, 11, 12, and 17 respectively). Even less important were found to be those to education, growth and industry (1/3 for SDGs 4, 8 and 9). Finally, forest contributions to gender and equality were assessed as secondary for Switzerland.

At international level too, forests are perceived to play a very important role in producing energy (SDG 7). Yet, very differently from Switzerland: they are considered as equally important (2.33/3) to alleviating poverty (SDG 1) and to fostering partnerships (SDG 17). The important contribution of global forests to poverty reduction was linked in priority to the provision of NTFPs and the regulation of natural cycles. Forests are further seen to play a prominent role for cities (SDG 11) and, in a slightly lighter manner, for hunger (SDG 2) and health (SDG 3). Contributions to growth, SPC and peace are classified as important but not highly ranked, while contributions to education, industry, equality and oceans were ranked at the low level 1/3.

### Interactions amongst FES

**Fig. 3:**
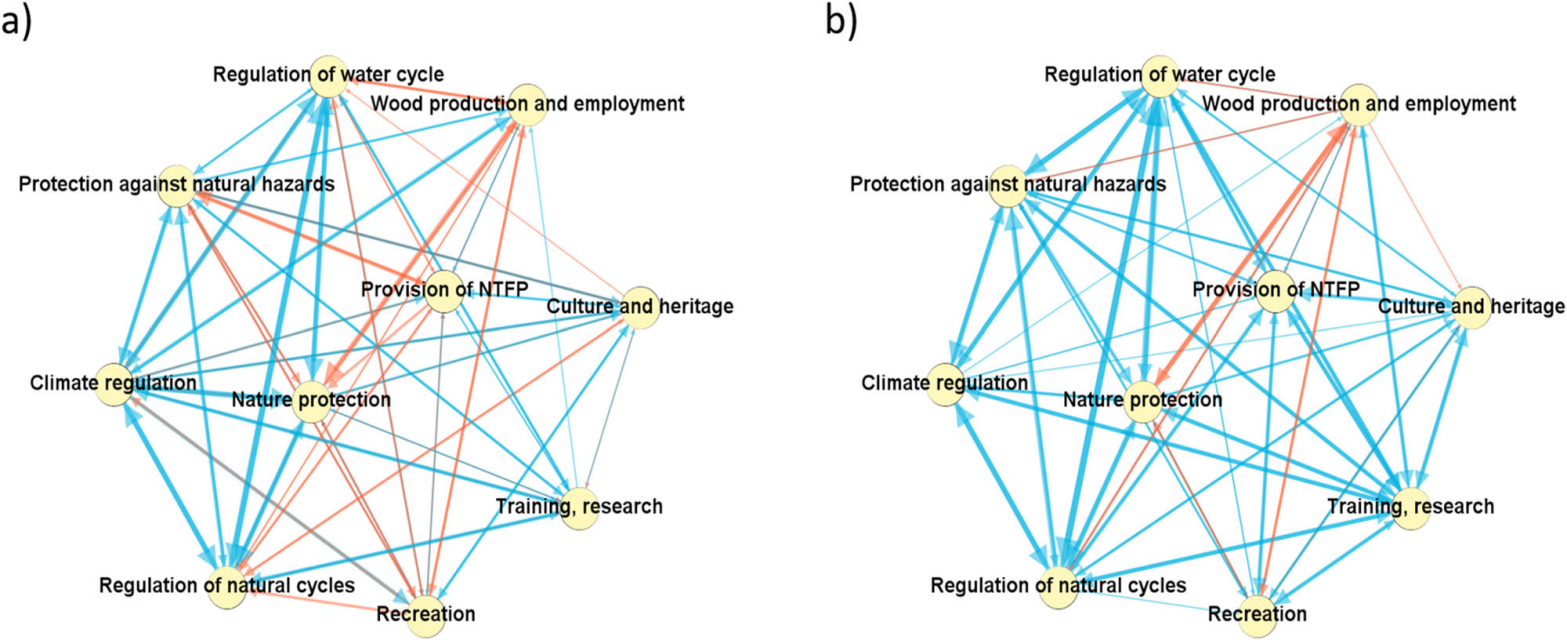
Interactions among FES in the Swiss (**a**) and international (**b**) contexts as revealed by the expert assessment. The arrow thickness corresponds to the weight of the given interaction, that is, the number of times an expert assessed it as a co-benefit (blue) or as a trade-off (red) interaction

Figure 3 displays the ten FES, interconnected as a network of the trade-offs (in red) and co-benefits (in blue) at Swiss and international levels. It is important to note that the interactions that were assessed context-dependent or irrelevant were not included in the analysis. The results we obtained from the assessment of interactions between these ten FES permitted to suggest some interpretations together with the group of experts. Based on Fig. 3, the following joint understanding emerged:

Importance of systems regulation and climate: Climate regulation was assessed as highly interconnected, particularly so in the Swiss context, where we argue it can play a multiplier effect of positive impact to other forest areas (Pfund and Pham-Truffert 2022). The other regulating services (water, natural cycles) were also assessed as highly connected in the network, and particularly in the international context.

Trade-offs between resource use and protection: In both contexts, the most striking trade-off was observed between nature protection and wood production and employment, suggesting the fundamental challenge of using forests while preserving them (Aggestam et al. 2020; Angst 2012).

Better definition and understanding of the Swiss context: More trade-offs were assessed in the Swiss context, which led to a discussion that a better contextualization effort has been possible there: Indeed, the experts in charge of assessing the international context had different backgrounds and experiences in potentially very diverse types of forests. The availability of socioeconomic information may play a significant role as well. A regular socioeconomic survey is conducted in Switzerland and informs about local people’s perceptions (Hegetschweiler et al. 2022).

Importance of training and NTFPs globally: FES such as training and the provision of NTFP appeared to be very important in an international context while not significant in Switzerland.

## Discussion

### Applied Method

Despite being based on a given ecosystem, directly linking FES to the SDGs pushed participating actors to avoid a siloed sectoral or disciplinary lens. Indeed, the process allows aligning ecosystem-based perceived realities to the different policy domains covered by the SDGs. From food security, to energy provision, to education and social equality, it goes beyond the nature conservation and human development paradigm. Tested at an abstract level, we can only assume that our approach is reproducible in concrete empirical case studies, notably to embrace their complexity (Freeman et al. 2015). During the final workshop, the experts with the most field experience stressed the potential to apply the method for spatially defined assessments toward informed decision making, and further monitoring. It cannot be seen as a standalone method, but could link in a structured way a multidisciplinary landscape assessment (such as initiated by Sheil et al. 2003) to broader decision-making levels. Indeed, the approach effectively enables the actors involved to have a basis to discuss toward solving conflicts. The semi-quantitative approach was tested at a relatively abstract level, but can be used for more concrete and context-specific case studies to map the perceptions of local actors

Our approach would have gained further legitimacy if we had involved early-on the experts in the pre-selection process of forest services potentially contributing to the SDGs. Yet, the ranking process itself was successful in obtaining a rapid overview of participants’ perceptions of the contributions of forests to SDGs. The evaluation of the interactions by pairs of forest services was experienced as being much more challenging by the participants. Issues may come from the theoretical generalization of FES and from the fact that ”all is context-dependent”. Thanks to the quantitative aspects collected through the surveys, the visualizations resulting from our analysis helped to nuance this context-dependency issue by highlighting common and diverging perceptions, in order to account for the most key trade-offs and synergies to tackle.

In our study, the relatively small sample of experts probably over-evaluated the importance of outlying answers, especially in terms of trade-offs. Further investigation was realized with the group of experts to identify ways of improving the process. Personal or group subjectivities did not represent a methodological issue as they were intended to appear and serve the dialog. Nevertheless, the workshop was necessary to be sure that a common understanding (and not a common perception) had been reached amongst all.

### Study Contribution

Multi-criteria decision analysis (Wolfslehner and Seidl 2010) and forest model simulations (Mina et al. 2017) are data-driven methods that have proved useful to support forest management while accounting for different FES. Yet, forest issues often remain discussed amongst forest actors and the linkages between forests and SDGs are hardly mentioned. In line with Timko et al. (2018), we believe that SDG, forest and land use planning experts can develop synergies on ways to ensure policy coherence and a proper involvement of decentralized levels. From a process point of view, as well as from an applied research perspective, adaptive and multifunctional forest planning can be integrated in a landscape (or land use) jurisdictional scale with other land-related dynamics.

In other situations where siloes need to be overcome, our method could help gather people and integrate forest management and land use issues in a new planning framework that would be more open (in terms of integrating FES in a broader spatial level, i.e. the landscape one for applied processes) and thus probably more participatory and adaptive. The integration of forest issues in domains that are “by essence” cross-sectoral, such as economy, land use planning, statistics and especially sustainable development, could have a rebound effect when the latter domains’ principles are followed by sectors more powerful than the forest one. In addition, a link to broader spatial scales and sustainable development could benefit from the current active trends of knowledge generation and initiatives for sustainable development in Switzerland and beyond.

We stress three main theoretical principles for an integrated landscape planning or management approach. Acknowledging multiple scales, functions and actors (Sayer et al. 2013), we encourage to (1) seek an understanding of the context, including cross-scale interdependencies, (2) involve the relevant actors, and (3) map their perceptions of the different land-uses and potential co-benefits or conflicts arising from them. We argue that understanding multi-scale processes in space and time (context), the different range of goods and services that a specific landscape can provide (land uses), and the various objectives of the different perspectives (actors), are all key aspects influencing landscape management outcomes. We therefore encourage to reproduce our approach in more concrete empirical case studies, in order to seek a better understanding of the given context, the relevant actors, as well as their different potential land uses or priorities.

The suggested methods are relatively easy to implement through a well facilitated exchange process. They need an excellent field-based and contextual knowledge but help define an informed ”snapshot” and launch dialog, and could also be used as a light monitoring tool as well. Forest and land use planning could in some cases act as a lever for sustainable development planning at various scales. Increased linkages between forest, land use planning and sustainable development planning could certainly help involve the population and decision-makers and demonstrate FES and their effects on the broader needs for sustainable development. The awareness of the SDGs (and the systemic nature of sustainable development) is widespread to most of the population, as exemplified by the 2030 Agenda, which has been formulated at all governance levels, including local communities. In line with Reed et al. (2022), such a method could help formulate pathways of a contextual theory of change to operationalize integrated landscape approaches.

## Conclusion

In Switzerland, policies in reaction to natural and economic issues have directly driven forests’ sustainable management and an increasing integration of various FES. The willingness to yield all the potential benefits from forests (from wood production to natural hazard protection, to societal functions) has led to diverse planning methods and silvicultural practices targeting multifunctionality. Nowadays, forest functions have been prioritized and mapped at the decentralized cantonal level. The integration of wood production and conservation has been particularly studied and practiced (Krumm et al. 2020). Knowledge-to-action networks between Swiss foresters have been fruitful recently, for instance, in anticipation of adaptation needs (Jenni et al. 2022).

Actual participatory approaches have been implemented since the 90 s in regional forest planning processes, but it always remained conditional to the State foresters and forest owners’ willingness. While this cross-sectoral tradition exists in the forest sector and the vision continues to promote and sustain forest multifunctionality (FOEN 2021), policy integration is not a generalized practice in other policy areas. Indeed, the policy integration of forests into land use, climate or water strategies remains complex due to sectoral approaches on the one hand, and because of an issue prioritization shedding lights primarily on the main concerns of CO_2_ emissions and biodiversity loss (rather than on rewarding already relatively well provided services).

Global issues and climate change pushes us to think of transformational changes that reach beyond the forest or other ecosystem-based realms. The forest-related knowledge, which has brought rich lessons learnt and best practices, will now not only have to adapt to climate change through new silvicultural management but also to the complex needs of the local, landscape, regional as well as the global community through new processes.

The presented exploratory network graph (Fig. 3) helps demonstrate general systemic perceptions, such as, in our FES example, the growing importance of forest contributions to climate change and the prevalence of regulating services over provisioning services, including wood production. Interactions illustrated interesting and sometimes unexpected outputs, notably the role that forest ecosystem services could play for partnerships at various scales.

The present study shows that participatory processes coupled with semi-quantitative methods can certainly help overcome siloes, including the forest sector one, and facilitate dialog amongst various actors. Our approach is based on integrated approaches to ecosystem services and rankings from actors (i.e., FES’ relative importance). It can be applied in various contexts and scales, as well as at various moments in time. The advantages of such an agile method outweigh the uncertainties related to subjective evaluations. With regard to rapid changes, decisions have to be made on an informed, transparent and shared basis. Decisions have in essence a part of subjectivity and risks, but need to be made.

Based on a forest example but possible to expand to other contexts, such combined approaches could serve as models or frames for complementary strategies in the face of climate change and in anticipating the future. In view of the uncertainties we are facing, the described systemic approach could support participatory prioritizations of pathways of change. Nevertheless, such an approach will remain dependent on effective cross-sectoral policy coordination at national level as well as an overall ”post-2030” systemic framework that would allow such an ecosystem-based analysis to be integrated in an internationally (and nationally) shared cross-sectoral vision of a desirable future.

### Supplementary Information

Supplementary_Material
